# A diaryl urea derivative, SMCl inhibits cell proliferation through the RAS/RAF/MEK/ERK pathway in hepatocellular carcinoma

**DOI:** 10.3389/fphar.2025.1605515

**Published:** 2025-07-10

**Authors:** Yue Fu, Weiyue Fang, Fuqiang Qiu, Juncai Lai, Yangjin Xu, Bin Chen, Yang Li, Xiaohui Zhu

**Affiliations:** ^1^ School of Pharmacy, Shenzhen University Medical School, Shenzhen University, Shenzhen, Guangdong, China; ^2^ College of Pharmacy, Shenzhen Technology University, Shenzhen, China; ^3^ Shenzhen ChemStrong Scientific Co., Ltd., Shenzhen, China; ^4^ Reference Material R&D Center, Shenzhen Jiangchuan Pharmaceutical Technology Co., Ltd., Shenzhen, China

**Keywords:** hepatocellular carcinoma, diaryl urea derivative, cell proliferation, RAS/RAF/MEK/ERK, bioactive compound

## Abstract

**Introduction:**

Hepatocellular carcinoma (HCC) ranks among the three most prevalent cancer-related diseases in terms of incidence. Hence, exploring drugs for HCC therapy is of great significance. Compounds with a diaryl urea structure have been reported to exhibit a broad range of biological activities, including anticancer activity. This study focuses on the specific diaryl urea derivative 4-(4-(3-(2-chloro-3-(trifluoromethyl)phenyl)ureido)phenoxy)-N-methylpicolinamide (SMCl), with particular emphasis on investigating its therapeutic effects against hepatocellular carcinoma (HCC) and elucidating the underlying molecular mechanisms.

**Methods:**

*In vitro* anti-cancer effects of SMCl were evaluated in HCC cell lines using MTS, colony formation, and wound healing assays. Western blot analyzed RAS/RAF/MEK/ERK pathway modulation. *In vivo* efficacy was assessed using a xenograft model.

**Results:**

The MTS and colony formation assays demonstrated that SMCl significantly decreased the viability of HCC cells. Western blot analysis demonstrated that SMCl effectively suppressed hepatocellular carcinoma proliferation by markedly inhibiting the RAS/RAF/MEK/ERK signaling pathway, with this inhibitory effect exhibiting both time- and concentration-dependent characteristics. SMCl also demonstrated significant therapeutic efficacy in the xenograft tumor model, achieving a tumor inhibition rate of 72.37%. Notably, it showed no significant impact on spleen weight or body weight in mice, indicating low toxicity to normal tissues.

**Conclusion:**

This study first elucidates the effects of SMCl on HCC cells and its impact on the RAS/RAF/MEK/ERK signaling pathway, providing a potential active compound for the clinical treatment of liver cancer.

## 1 Introduction

At present, hepatocellular carcinoma (HCC) ranks as the fourth leading cause of cancer-related mortality globally. Especially in less developed regions of Africa and Asia, the death toll attributed to this disease is predicted to rise sharply in the next decade (Siegel et al., 2024). Chronic infections caused by hepatitis B or C virus, nonalcoholic steatohepatitis due to obesity (with or without type 2 diabetes), and excessive alcohol consumption are key risk factors for HCC (Alqahtani et al., 2019; Reddy et al., 2023). Additional risk factors encompass metabolic disorders like α1-antitrypsin deficiency, hemochromatosis, and autoimmune conditions. The intake of aflatoxin-contaminated foods and tobacco usage also have a notable impact on the progression of this disease. The variation in risk exposure is broad, depending on geographic and sociocultural contexts (Ducreux et al., 2023). Despite available surgical and transplantation approaches that offer the best outcomes, only about 15% of patients are eligible for these potentially life-saving measures, as the majority are diagnosed at advanced stages of the disease (Anwanwan et al., 2020; Terrault et al., 2023).

Chemotherapy remains one of the primary treatment modalities for HCC, mainly due to its significant effectiveness in rapidly diminishing tumor burden. HCC is often characterized by large tumor masses or concurrent liver dysfunction, which makes patients ineligible for curative approaches such as surgery, radiotherapy, or immunotherapy. In such cases, chemotherapy employs cytotoxic agents to swiftly kill cancer cells or inhibit their proliferation, leading to substantial tumor shrinkage and alleviation of clinical symptoms, thereby creating opportunities for subsequent treatments (Kew, 2011). Moreover, chemotherapy plays a crucial role in various combination therapy regimens, such as those involving targeted therapies or immunotherapies, enhancing overall therapeutic efficacy. Despite its limitations, including toxicity and the emergence of drug resistance, chemotherapy remains indispensable in the management of HCC, particularly in settings where therapeutic resources are constrained, or alternative options are unavailable. Chemotherapeutic agents are affordable and have broader applicability, making them especially vital for patients in low- and middle-income countries (Ocran Mattila et al., 2021). Introduced in 2007 for treating advanced inoperable HCC, Sorafenib, a multikinase inhibitor that halts cell proliferation and angiogenesis, represented a breakthrough (Kim et al., 2017). Subsequently, other multi-kinase inhibitors like Lenvatinib have received approval and are found to be equally effective as Sorafenib for initial treatment. Drugs like Regorafenib, Cabozantinib, and Ramucirumab have been approved as secondary agents to increase survival rates. Although new immunotherapies for HCC have offered new hope, their overall effectiveness is still limited (Yang et al., 2023; Bicer et al., 2023). Identifying novel candidate compounds suitable for clinical development in HCC management persists as a vital research imperative.

Diphenyl urea derivatives possess significant application potential due to their unique structure and diverse biological activities. Structurally, the core of diphenyl urea derivatives consists of a urea group (-NH-CO-NH-) linking two aromatic rings (Ladd et al., 2024). Various substituents (such as halogens, alkyl groups, amino groups, etc.) can be attached to the aromatic rings, allowing for modulation of the molecule’s electronic effects and spatial configuration, which in turn affects its biological activity and selectivity. This structural characteristic endows the diphenyl urea derivatives with enhanced molecular stability and chemical tunability, facilitating the optimization of their pharmacological properties through structural modifications. Numerous diphenyl urea derivatives have been identified for their anticancer activity, with multi-kinase inhibitors being a prominent example. Sorafenib, a multi-kinase inhibitor, effectively inhibits tumor cell proliferation and angiogenesis, showing significant therapeutic effects in liver and kidney cancers.

Additionally, it has been reported that Sorafenib suppresses the growth and metastasis of hepatocellular carcinoma by blocking the activation of STAT3. Furthermore, certain diphenyl urea derivatives have demonstrated inhibitory effects on viral protein targets, including SARS-CoV-2 and influenza A virus (IAV) (Degirmenci et al., 2020). These derivatives interact with specific proteins involved in viral replication, reducing viral load and slowing down the disease progression. In the research of SARS-CoV-2, it has been found that diphenyl urea derivatives can inhibit the main protease (Mpro) and RNA-dependent RNA polymerase (RdRp), both of which are critical for viral replication. The diverse biological activities of diphenyl urea derivatives have made them a focal point in drug development.

The successful application of Sorafenib highlights the significance of diphenyl urea derivatives in cancer treatment. With ongoing research, more diphenyl urea derivatives with specific biological activities are expected to be discovered. An important aspect of cell communication is the RAS/RAF/MEK/ERK signaling network, which is responsible for regulating processes like cell proliferation, survival, and differentiation. Several natural agents, including alkaloids, phenolics, terpenoids, and nano-formulations, impact the Ras/Raf/MAPK route (Gravandi et al., 2023). This pathway also manages signals from complex cell networks to regulate cellular activities. Although researchers have known the essential elements of the MAPK pathways for nearly four decades, fully understanding the intricate molecular dynamics that govern these pathways remains a challenge. Intense efforts have been made to control Raf, particularly after identifying drug resistance and unexpected activation upon the attachment of inhibitors to the kinase (Imoto et al., 2023). Phosphorylation and conformational changes, such as autoinhibition and dimerization, are involved in Raf regulation. A few RAS/RAF/MEK/ERK route inhibitors have demonstrated potential in the treatment of liver cancer in clinical trials (Spencer-Smith and Morrison, 2024; Zinatizadeh et al., 2019). As a cancer treatment strategy, dual inhibition of this pathway is highly effective (Cheng et al., 2019).

In this study, we have discovered a new active compound, 4-(4-(3-(2-chloro-3-(trifluoromethyl) phenyl) ureido) phenoxy)-N-methylpicolinamide, which bears structural resemblance to sorafenib. This compound shares the aminoquinoline backbone, an aromatic ring, and fluorine atoms with sorafenib. The aromatic ring provides hydrophobicity, while the fluorine atoms contribute to enhanced membrane permeability and metabolic stability. The key difference is that sorafenib has a chlorine atom at the para-position of the aromatic ring, whereas in 4-(4-(3-(2-chloro-3-(trifluoromethyl)phenyl) ureido) phenoxy)-N-methylpicolinamide, the chlorine atom is situated between the aromatic ring and the trifluoromethyl group. We named this new compound “sorafenib meta-chlorine” and abbreviated it as SMCl. This study aims to evaluate the efficacy of SMCl, a new RAS/RAF/MEK/ERK inhibitor, in treating HCC through cell and animal model experiments.

## 2 Materials and methods

### 2.1 Materials

#### 2.1.1 Cell culture

The Huh7, Hep3B, PLC/PRF/5 and SMMC7721 cell lines were cultured in DMEM (Dulbecco’s Modified Eagle Medium, Gibco, United States) supplemented with 10% fetal bovine serum (ExCell Bio, China) and 1% penicillin-streptomycin solution (100 U/mL each, CellorLab, China). SMMC7721 cell line was purchased from the American Type Culture Collection (ATCC). Hep3B, PLC/PRF/5 and Huh7 cell line were obtained from HyCyte (https://www.hycyte.com/; Catalogue No. TCH-C195, No. TCH-C298 and No. TCH-C217). The culture medium was kept at 37°Cin an incubator with 5% carbon dioxide (Ding et al., 2015).

#### 2.1.2 Chemicals

DMSO (Sigma-Aldrich, United States) was used to dissolve SMCl, which was supplied by the QCS Reference Material Research and Development Center in China. We conducted a qualitative analysis of SMCl, and the results are shown in the Supplementary Figures S1–S8. Sorafenib (Shanghai Macklin Biochemical, China); Antibodies against Ras, Raf, pRaf, and GAPDH(Abcam, United Kingdom); Rabbit anti-mouse IgG-HRP conjugates, p21, Erk, and pErk (Cell Signaling Technology, United States); All antibodies were utilized for Western blot analysis. The [3-(4,5-dimethylthiazol-2-yl)-5-(3-carboxymethoxyphenyl)-2-(4-sulfophenyl)-2H-tetrazolium(MTS)and phenazine methosulfate(PMS) were provided by Promega Corporation in the US. The MTS and PMS powders are individually dissolved in Dulbecco’s Phosphate-Buffered Saline (DPBS), sterilized, and stored at −80°C. Beyotime Biotechnology in China is responsible for the production of the EdU Cell Proliferation Kit.

### 2.2 Methods

#### 2.2.1 Evaluation of cell viability

Following the methodology described in a previous study (Wu et al., 2015), the MTS was applied to determine the viability of the cells during each experiment, Hep3B and Huh7 cells were seeded in 96-well flat-bottom plates at an appropriate concentration. The next day, the cells were subjected to a range of different concentrations of SMCl. Simultaneously, the control cells were given the same amount of vehicle (DMSO) as the cells that were treated with the drug. Following a treatment period of 72 h, Remove the compound-containing medium and replace it with the prepared solution (MTS:PMS ratio of 20:1), adding 100 μL per well to a 96-well plate. Incubate for 1–4 h, then determine the optical density at 490 nm.

#### 2.2.2 EdU test

Twenty-four well plates were used to seed the cells. Following the natural adhesion process, different concentrations of SMCl were added, specifically 0 μM, 2.5 μM, 5 μM, and 10 μM. The EdU test was utilized in accordance with the instructions supplied by the kit to determine the number of cells in the proliferation phase.

#### 2.2.3 Formation of colonies

The cell lines Hep3B and PLC/PRF/5 were cultured for a period of 48 hours after being placed into 12-well plates at a total of one thousand cells in each well. After that, the cells were subjected to SMCl over a period of 24 hours, with concentrations of 2.5, 5, and 10 μM. To encourage the formation of colonies, the cells were cultured for a span of 10 days, with medium changes occurring every 5 days. Finally, photographs were taken of the colonies (Luo et al., 2023).

#### 2.2.4 Wound healing test

In order to evaluate cell locomotion, the wound closure assay was utilized *in vitro*, as outlined in the previous section (Zhang et al., 2019). Every single experiment was carried out three times, and each experiment was replicated three times to ensure accuracy.

#### 2.2.5 Analysis of the western blot

Cells were transplanted into plates with six wells. Following 48 h of treatment with SMCl at graded concentrations (0, 2.5, 5, and 10 μM), cells were harvested for subsequent analysis. Another group of cells was treated with 10 μM SMCl for varying durations (0, 24, 36, and 48 h) before collection. The cells were lysed with RIPA lysis buffer that contained PMSF (Beyotime, China). Protein quantification was performed using a BCA assay kit (Beyotime, China). Proteins were separated by SDS-PAGE and transferred to PVDF membranes (Millipore, Canada). Membranes were blocked with 5% skim milk at room temperature for 1 h. After that, the membranes were subjected to an incubation procedure using primary antibodies that targeted Erk, pErk, and GAPDH (Cell Signalling Technology, Danvers, Massachusetts, United States), which was then followed by the application of secondary antibodies that were appropriate for the situation. The use of enhanced chemiluminescence (ECL) detection reagents allowed for the visualization of protein bands (Zhang et al., 2023).

#### 2.2.6 The study of animals

The development of a subcutaneous tumor model was carried out in order to gain an understanding of the effects that SMCl has on the growth of tumors in living organisms (Kim et al., 2019). The Guangdong Provincial Medical Experimental Centre provided the female BALB/c mice that were used in this study. These mice were aged between four and 5 weeks and were housed in the SPF Laboratory Animal Centre of Shenzhen Rongwan Biotechnology Co., Ltd. When carrying out procedures that involved animals, the Rongwan Biological Laboratory Animal Center strictly adhered to the regulations that had been established for the welfare of laboratory animals and ethics concerning laboratory animals with the reference approval number RW-IACUC-24-0022. SMMC7721 cells, with a concentration of 1 × 10^^6^, were administered to each mouse through a subcutaneous injection. The injection was administered in the right flank of each mouse. Beginning on the day that the cell injection was administered, measurements of the body weight and the volume of the tumor were taken every 3 days. For determining the volume of the tumor, the formula that was utilized was (length × width^2^)/2. The mice were randomly divided into two groups, each consisting of five to seven mice, once the tumors had grown to a volume of 50 mm squared. This was done after the tumors had begun to grow. When cells were administered, the average amount of time it took for tumors to reach a volume of 50 mm cubic was approximately five to 6 days, with a standard deviation of three and a half percentage points. Included in the treatment groups were both the SMCl (50 mg/kg/day) group as well as the control group, which consisted of the drug vehicle. The administration of each treatment was carried out by means of oral gavage. Following the completion of the experiment, which lasted for a period of 3 weeks, the tumors were promptly removed after the euthanasia procedure was completed. Each tumor was dissected to obtain tumor tissue lysates for Western blot analysis. This was done to make the histological examination more manageable.

#### 2.2.7 Statistical analysis

The quantitative findings were obtained from at least three distinct trials, and the data is provided as the average value together with the standard deviation. The statistical analyses were conducted using GraphPad Prism Software, version 7.0 (GraphPad Inc., La Jolla, California, United States). We employed the student’s t-test to ascertain significant disparities between two groups. To evaluate the variation among various groups, either one-way or two-way ANOVA was utilized, along with Bonferroni’s correction for multiple comparisons.

## 3 Results

### 3.1 SMCl possesses substantial anti-proliferation effects on HCC cell lines

**FIGURE 1:**
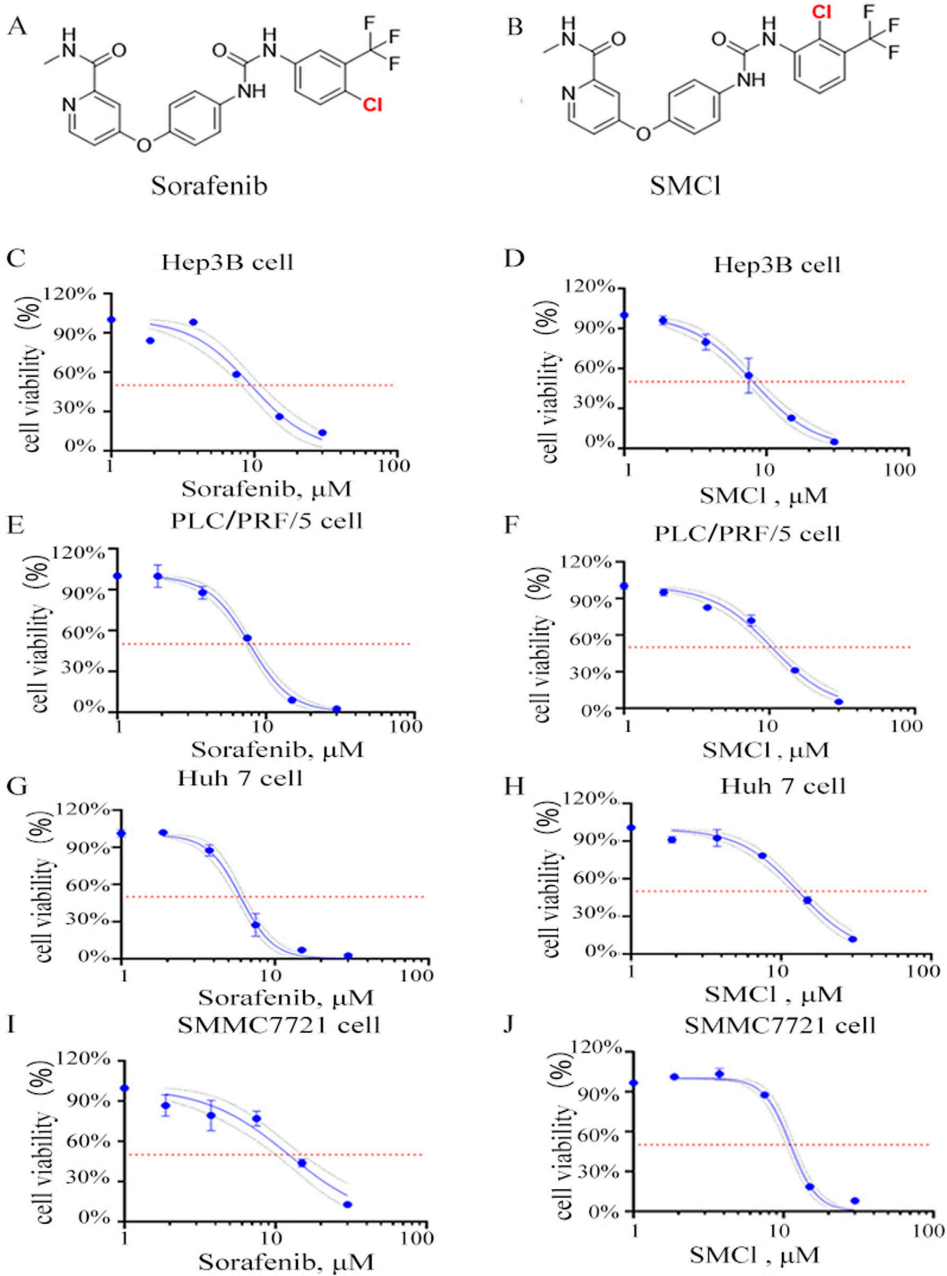
Effects of SMCl on the cell viability of HCC cells. **(A,B)** Chemical structure of SMCl and Sorafenib, **(C–J)** Effects of different concentrations of SMCl and Sorafenib on the proliferative ability of liver cancer cells at 72 h.

The structure of SMCl and its differences from sorafenib are shown in Figures 1A. Supplementary Figure S1 shows that the purity of SMCl is 98% as determined by HPLC. Supplementary Figures S2, S3 represent the ^1^H NMR and ^13^C NMR spectra of SMCl (Figures 1A,B). To further distinguish SMCl from sorafenib, we performed two-dimensional NMR analysis of SMCl, with Supplementary Figures S4–S8 presented respectively. To determine the potential inhibitory effect of SMCl on liver cancer cell growth, we first assessed its influence on cell proliferation and viability using the MTS assay in PLC/PRF/5 and SMMC7721 cells, using sorafenib as the positive control compound. As depicted in Figures 1C–J, SMCl demonstrated a concentration-dependent reduction in cell viability across Hep3B, PLC/PRF/5, and SMMC7721 cell lines. The IC_50_ values of SMCl for Hep3B, PLC/PRF/5, Huh7 and SMMC7721 cells were 8.033 μM, 10.37 μM, 12.98 μM and 11.83 μM, respectively and the IC_50_ values of sorafenib for Hep3B, PLC/PRF/5, Huh7 and SMMC7721 cells were 9.477 μM, 7.755 μM, 5.697 μM and 12.22 μM, respectively. These results indicate that SMCl exhibits inhibitory effects on different types of liver cancer cells. Specifically, the lowest IC_50_ value (8.033 μM) was observed in Hep3B cells, suggesting that SMCl has the most significant anticancer effect in this cell line and can effectively inhibit cell proliferation at lower concentrations. Although the IC_50_ values for PLC/PRF/5 and SMMC7721 cells are higher, at 10.37 μM and 11.25 μM respectively, these data still demonstrate that SMCl exerts a certain level of inhibition on liver cancer cells. Notably, compared to sorafenib, SMCl exhibits stronger effects on Hep3B and SMMC7721 cells, while the opposite is observed in Huh7 and PLC/PRF/5 cells. These experimental results highlight the broad activity of SMCl across different liver cancer cell lines, providing a theoretical basis for its development as a potential anti-liver cancer drug.

### 3.2 SMCl inhibits the migrative and colony forming capacities of HCC cells *in vitro*

**FIGURE 2:**
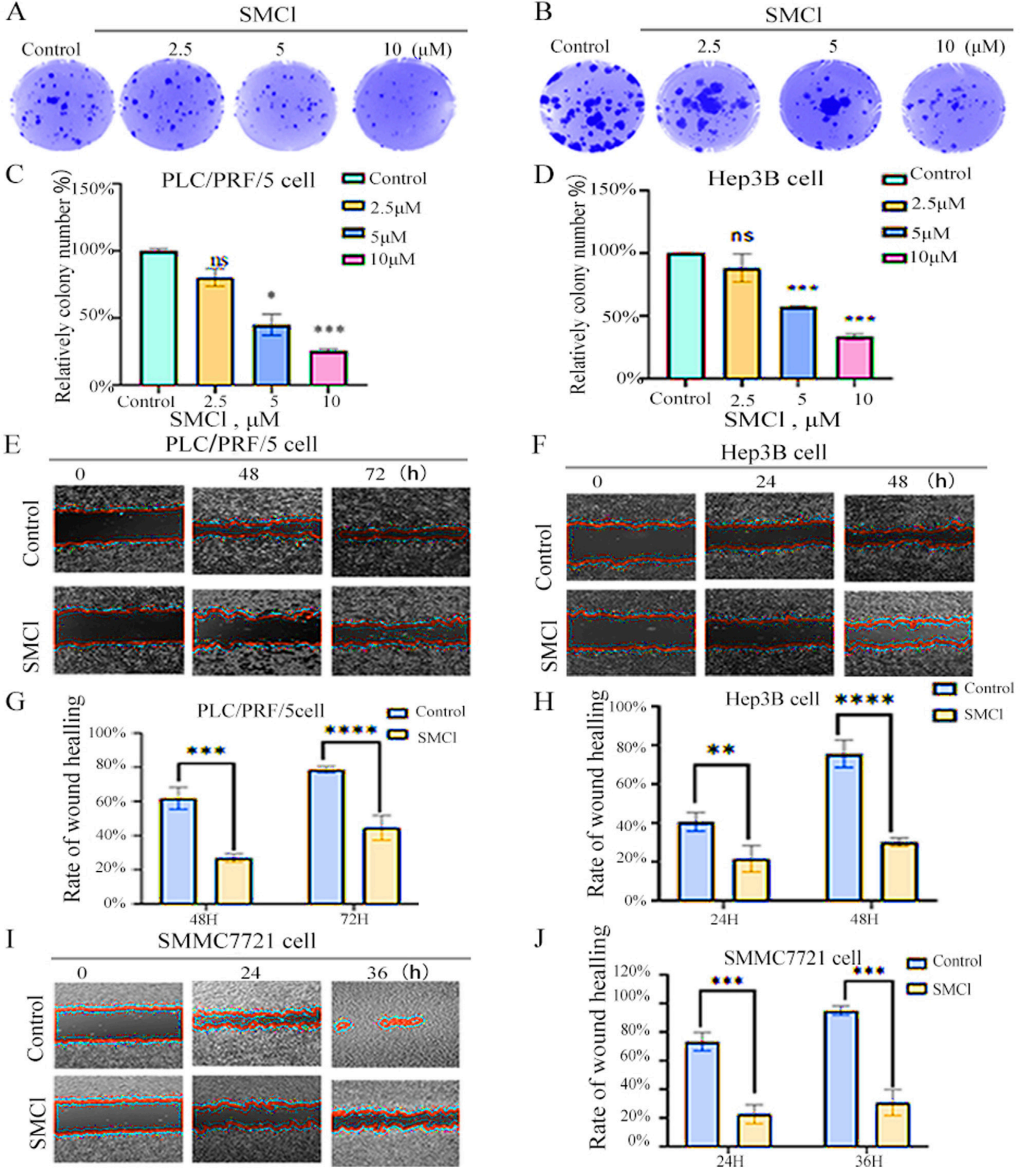
SMCl suppresses cell migration and colony formation. **(A,B)** Cells were treated with 0, 2.5, 5, and 10 μM SMCl for the indicated time. **(C,D)** Relative colony number at different concentration of SMCl. **(E,F,I)** Wound healing assay was performed. Cells were treated with 10 μM SMCl at 0, 24, 36, and 48 h. Scale bar, 100 μm. Experiments were independently conducted in triplicate. **(G,H,J)** The area of non-migrant cells was quantified using ImageJ software. Data are presented as mean ± SEM (*p < 0.05, **p < 0.01, and ***p < 0.001 vs. the control group).

For determining the effect that SMCl has on the migratory and colony-forming capabilities of hepatocellular carcinoma (HCC) cells, we conducted wound healing and colony-formation assays. The utilization of various concentrations of SMCl was shown to effectively impede the movement of HCC cells, as shown in Figures 2A–D. Based on this observation, it appears that SMCl exerts an effect that is dose-dependent in terms of its ability to inhibit the migration of hepatocellular carcinoma (HCC) cells, and the high concentration (10 μM) inhibited Hep3B colony formation by more than 50%. In addition, the wound healing assay provided further evidence that supports these findings (Figures 2E–J). More precisely, the study found that increased concentrations resulted in a significant reduction in the ability of HCC cells migration, particularly in SMMC7721 cells, this inhibitory effect on wound closure was more pronounced, showing over 60% inhibition at 36 h, as evidenced by SMCl. The results emphasize the ability of SMCl which has the capability to inhibit the migration and colony formation of HCC cells, suggesting that it may suppress the progression of hepatocellular carcinoma by affecting cell motility and proliferation. The development of HCC is not only dependent on the proliferation of tumor cells but also closely related to their migration and invasion capabilities. Cell migration, especially during cancer metastasis, is a key factor in determining tumor spread (Li et al., 2023). Therefore, the inhibitory effect of SMCl may indicate that it can interfere with the invasive behavior of tumor cells, limiting the spread of cancer cells to surrounding tissues and thus reducing the risk of liver cancer metastasis.

### 3.3 SMCl inhibits the growth of HCC cells via the RAS/RAF/MEK/ERK pathway

**FIGURE 3:**
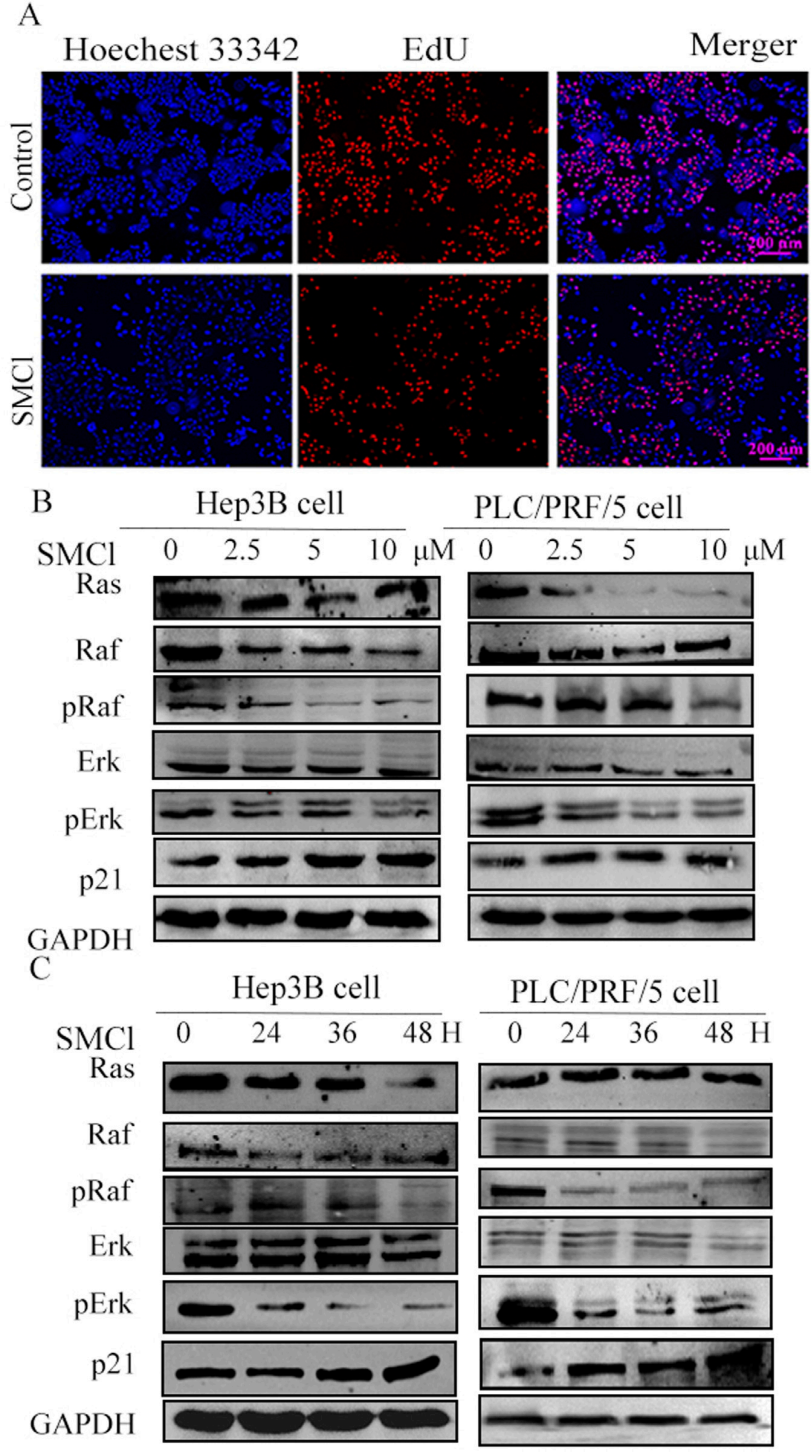
Investigated the impact of SMCl on the Ras/Raf/MAPK pathways in HCC cell lines. **(A)** Hep3B cells were divided into a control group and an SMCl treatment group (10 μM). After 48 h of treatment, fluorescence microscopy showed a marked reduction in the number of EdU-positive cells in the SMCl-treated group compared to the control group. **(B)** Effects of 0, 2.5, 5, and 10 μM SMCl treatment for 48 h on protein levels of Ras, Raf, Erk, and p21 in both Hep3B and PLC/PRF/5 cell lines. **(C)** Time-dependent effects of 10 μM SMCl treatment at 0, 24, 36, and 48 h on protein levels of Ras, Raf, Erk, and p21 in Hep3B and PLC/PRF/5 cells.

For the EdU experiment, we used Hep3B Cell lines divided into a control group and an SMCl treatment group (10 μM). After 48 h of treatment, fluorescence microscopy indicated that the SMCl treatment group had a much smaller number of EdU-positive cells than the control group (Figure 3A). These results indicate that SMCl can significantly inhibit DNA synthesis activity in Hep3B cells, suggesting its potential anti-tumor effect through inhibition of cell proliferation. Moreover, treatment of Hep3B and PLC/PRF/5 cells with increasing concentrations of SMCl resulted in dose-dependent downregulation of Ras, phosphor-Raf, and Erk protein levels (Figure 3B). Notably, SMCl reduced both phosphorylated and total forms of Raf and Erk, suggesting non-specific inhibition of the MAPK pathway. Time-course experiments demonstrated progressive decreases in Erk and Raf phosphorylation with prolonged SMCl exposure (Figure 3C), confirming time-dependent suppression of pathway activity. These findings collectively indicate that SMCl effectively inhibits the RAS/RAF/MEK/ERK signaling cascade through both concentration- and duration-dependent mechanisms. Concurrently, examination of the downstream proliferation-associated protein p21 revealed significant upregulation following SMCl treatment, mechanistically substantiating its anti-proliferative efficacy through cell cycle checkpoint activation.

### 3.4 SMCl inhibits tumor growth in xenograft mouse models

**FIGURE 4:**
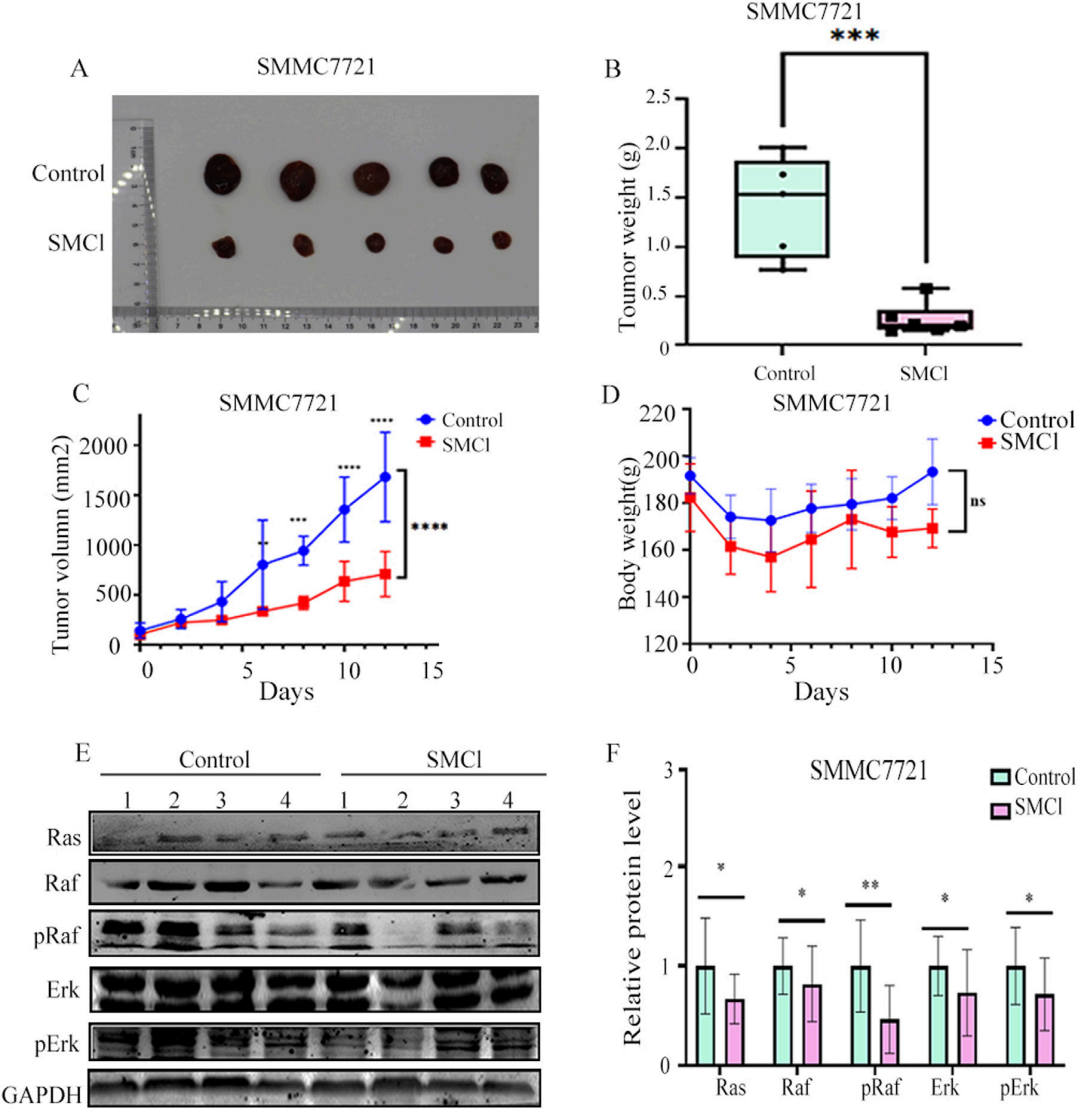
SMCl inhibits tumor progression in the SMCC7721 xenograft model. **(A)** Nude mouse bearing xenografted tumors were randomly divided into two groups: one as the control group treated daily with DMSO, and the other treated with SMCl 50 mg/kg/day for 12 days. Dissected tumors were obtained from mice in both the control and SMCl groups. **(B)** Tumor weight of subcutaneous tumors from the control and SMCL groups. **(C)** Comparison of subcutaneous tumor volumes from the control and SMCL groups (n = 5). **(D)** Comparison of body weight of nude mice from the control and SMCL groups. **(E,F)** Protein levels of pRaf, pErk, and Ras in subcutaneous tumors from the DMSO and SMCL groups. *p < 0.05, **p < 0.01, ***p < 0.001, ****p < 0.0001.

We investigated whether SMCl could reduce tumor growth in a CDX mice model. As demonstrated in Figures 4A–C, SMCl (50 mg/kg) significantly inhibited the growth of the subcutaneous SMMC7721 tumor and compared to the control group, the tumor inhibition rate reached 72.37%. Notably, body weight was not significantly different between the SMCl group and the control group mice (Figure 4D). Western blotting revealed the same trend of associated protein changes (Figures 4E,F). The results of all animal experiments indicate that SMCl can effectively inhibit tumor growth while having no significant effects on the animals themselves, indicating low toxicity and side effects, which suggests good biological safety. Low toxicity is a critical feature in cancer treatment, as it reduces damage to normal tissues and enhances the safety and tolerability of treatment (Lou et al., 2015). Additionally, through protein extraction from animal tissues and Western blot experiments, the study also confirmed the effect of SMCl on the RAS/RAF/MEK/ERK signaling pathway. This pathway plays a key role in the development of liver cancer and other tumors by regulating processes such as cell proliferation, differentiation, and migration, promoting tumor growth and metastasis. Therefore, Impact of SMCl on this pathway further suggests that it may inhibit tumor progression by interfering with critical signaling pathways in tumor cells. These animal study results provide strong evidence of SMCl biological activity *in vivo* and support its potential as a therapeutic agent for liver cancer.

## 4 Discussion

**FIGURE 5:**
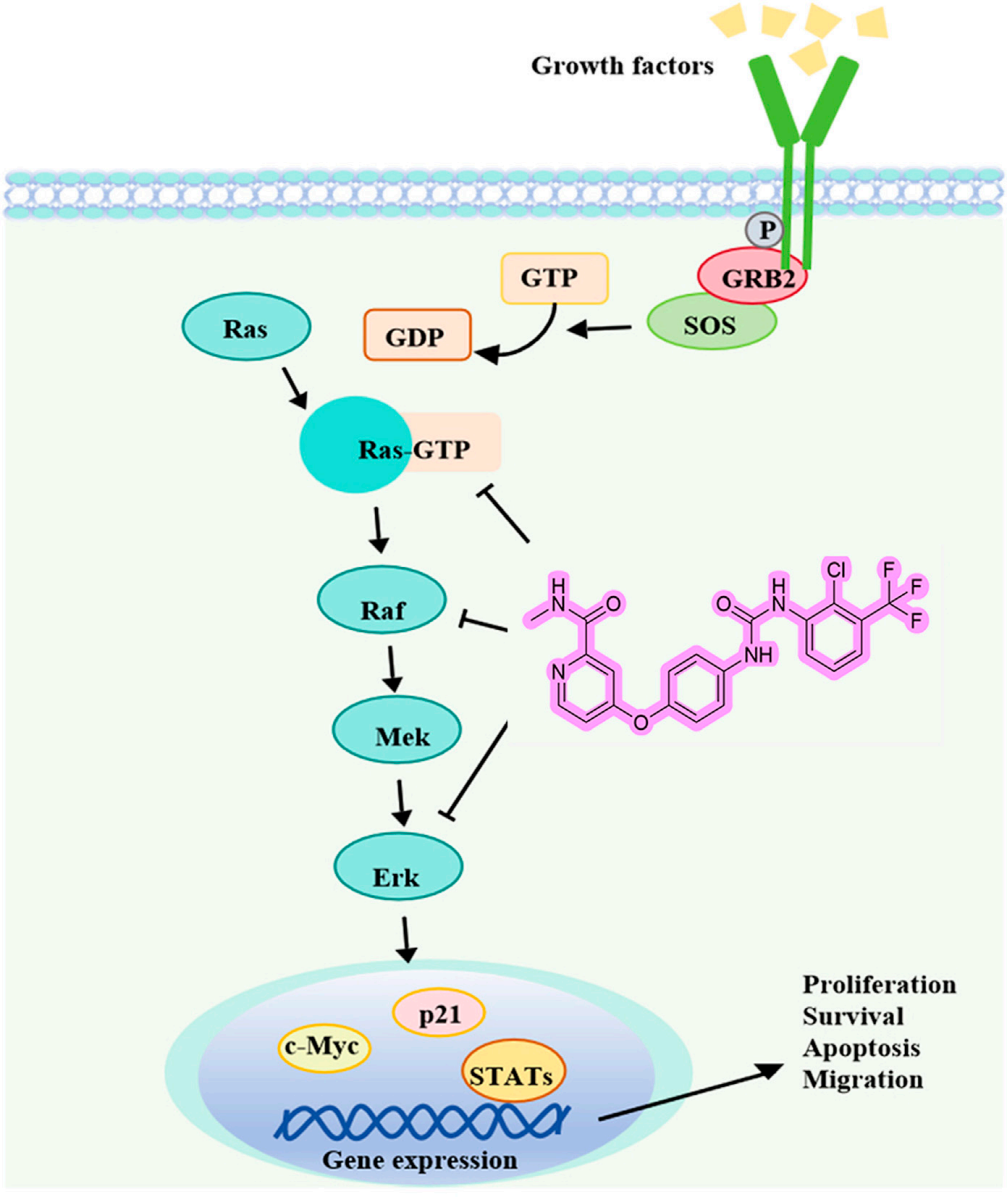
SMCl exerts anti-HCC effects by targeting the RAS/RAF/MEK/ERK pathway.

Liver cancer is characterized by high mortality and incidence rates, while the scarcity of clinical therapeutics remains a persistent challenge. The discovery of potential candidate compounds is therefore of critical importance. With diaryl urea as the core of anticancer molecules, researchers can develop more potent drugs that precisely attack cancer cells while leaving healthy cells unharmed (Wang et al., 2020; Liu et al., 2016). SMCl is a diaryl urea derivative, and this study represents the first evaluation of its antitumor effects on Hep3B and SMMC7721 liver cancer cells, demonstrating its potential as a promising therapeutic agent. These initial findings have been further supported by subsequent cell and animal experiments, providing solid evidence of SMCl efficacy in the treatment of HCC. Furthermore, our study revealed that SMCl exhibits the characteristic biological activity of diaryl urea derivatives, effectively inhibiting the activity of several key intracellular kinases, including RAF, RAS, and ERK kinases. The mechanism of SMCl is illustrated in Figure 5.

RAF and ERK kinases play critical roles in tumor cell proliferation, migration, and angiogenesis, with aberrant kinase activity being a major driving force in tumor development and progression (Garuti et al., 2016). By inhibiting multiple kinase activities, SMCl exerts its antitumor effects through the RAS/RAF/MEK/ERK signaling pathway (Bahar et al., 2023). As a consequence, the primary signaling pathway targeted by multi-kinase inhibitors is the mitogen-activated protein kinase (MAPK) pathway, specifically the RAS/RAF/MEK/ERK cascade. Raf kinase plays a crucial role in this pathway by sequentially activating downstream targets like Mek and Erk, regulating essential cellular processes such as development, cell cycle control, multiplication, specialization, and longevity (Scardaci et al., 2024). An imbalance in this pathway has been tied to several diseases, with cancer being one of them. The story of Raf inhibitors begins with sorafenib, a drug initially aimed at targeting Ras-driven tumors. Unfortunately, it was insufficient as cancer cells developed resistance to the treatment (Wei et al., 2023; Liu et al., 2023). SMCl, as a diaryl urea derivative structurally similar to sorafenib, has demonstrated comparable safety and therapeutic efficacy (Ghannam et al., 2023; Ommi et al., 2023). Interestingly, SMCl is often produced as a byproduct during the manufacturing process of sorafenib. Due to the structural similarity between SMCl and sorafenib, scientists initially expressed concerns that this byproduct might interfere with sorafenib’s mechanism of action to some extent, potentially diminishing its efficacy. In cancer treatment, the potency and specificity of a drug are critical to its clinical outcomes, which made this potential impact a matter of widespread attention. However, our study revealed findings that were contrary to these initial concerns—SMCl not only did not compromise sorafenib’s efficacy but also demonstrated biological activity similar to that of sorafenib. This discovery suggests that SMCl exerts its anticancer effects through mechanisms akin to those of sorafenib, inhibiting the proliferation and migration of tumor cells. Furthermore, as a byproduct of sorafenib production, SMCl has the advantages of being relatively cost-efficient and having a stable supply, which provides significant economic and technological benefits for its future large-scale production and drug development. In summary, these findings not only offer new insights into the mechanism of action of sorafenib but also lay a solid foundation for the development of novel anticancer drugs with enhanced efficacy and reduced side effects.

However, our study has certain limitations that need to be addressed in future research. Firstly, although experimental results indicate that SMCl effectively reduces Ras expression, its precise molecular mechanism remains unclear. It is still unknown whether SMCl directly binds to Ras to inhibit its activity or indirectly regulates upstream or downstream signaling molecules to achieve this effect. Addressing this question will not only help elucidate the mode of action of SMCl but also provide insights for optimizing its structure. Secondly, our current evaluation of SMCl antitumor effects is primarily based on HCC cell lines and xenograft tumor models, resulting in a relatively narrow scope of the research. HCC is a highly heterogeneous disease, with tumors in different patients exhibiting significant differences in genetic mutation profiles, microenvironments, and drug sensitivities (Suresh and Dhanasekaran, 2022; Safri et al., 2024). Beyond this, sorafenib resistance has been well documented, our study did not investigate whether SMCl remains effective against sorafenib-resistant strains. However, the differential responses of various HCC cell lines to SMCl versus sorafenib warrant further exploration. Therefore, in subsequent studies, we will conduct a comprehensive comparison of the mechanistic differences between sorafenib and SMCl at deeper molecular levels, thereby exploring potential solutions to sorafenib resistance and incorporate more diverse vivo models, such as patient-derived xenografts or other HCC subtypes, to comprehensively assess SMCl antitumor activity. Additionally, studies should explore the potential of SMCl in combination therapies, such as with existing targeted drugs or immunotherapies (Vanneman and Dranoff, 2012), to evaluate its synergistic effects. Lastly, although our study preliminarily validated the safety and efficacy of SMCl in cell and animal models, clinical trials are a critical step in translating it into clinical applications. Future clinical trials need to evaluate not only the efficacy of SMCl in HCC patients but also its pharmacokinetic properties, safety, and tolerability. Furthermore, studies should investigate the therapeutic effects of SMCl in patients at different stages of liver cancer and explore its compatibility and applicability with existing treatment regimens.

## Data Availability

The original contributions presented in the study are included in the article/Supplementary Material, further inquiries can be directed to the corresponding authors.

## Supplementary material

SUPPLEMENTARY FIGURE S1 High-Performance Liquid Chromatography (HPLC) chromatogram of SMCl, with a retention time of 8.003 min and a purity of 98%.

SUPPLEMENTARY FIGURE S2 Proton Nuclear Magnetic Resonance (^1H NMR) spectrum of SMCl, with chemical shifts and corresponding protons annotated.

SUPPLEMENTARY FIGURE S3 Carbon-13 Nuclear Magnetic Resonance (^13C NMR) spectrum of SMCl.

SUPPLEMENTARY FIGURE S4 Distortionless Enhancement by Polarization Transfer (DEPT-135) NMR spectrum of SMCl.

SUPPLEMENTARY FIGURE S5 Correlation Spectroscopy (COSY) NMR spectrum of SMCl, showing proton-proton coupling relationships with cross-peaks indicating correlations.

SUPPLEMENTARY FIGURE S6 Nuclear Overhauser Effect Spectroscopy (NOESY) NMR spectrum of SMCl, displaying spatial correlations between protons, with significant cross-peaks indicating close spatial proximity.

SUPPLEMENTARY FIGURE S7 Heteronuclear Single Quantum Coherence (HSQC) NMR spectrum of SMCl, showing direct one-bond correlations between ^ 1H and ^ 13C nuclei.

SUPPLEMENTARY FIGURE S8 Heteronuclear Multiple Bond Correlation (HMBC) NMR spectrum of SMCl, illustrating long-range ^ 1H-^ 13C correlations.
