# Supplementary figures and images for: A diaryl urea derivative, SMCl inhibits cell proliferation through the RAS/RAF/MEK/ERK pathway in hepatocellular carcinoma
Source: Front Pharmacol. 2025 Jul 10;16:1605515. doi: 10.3389/fphar.2025.1605515 (PMC12287612; doi:10.3389/fphar.2025.1605515)

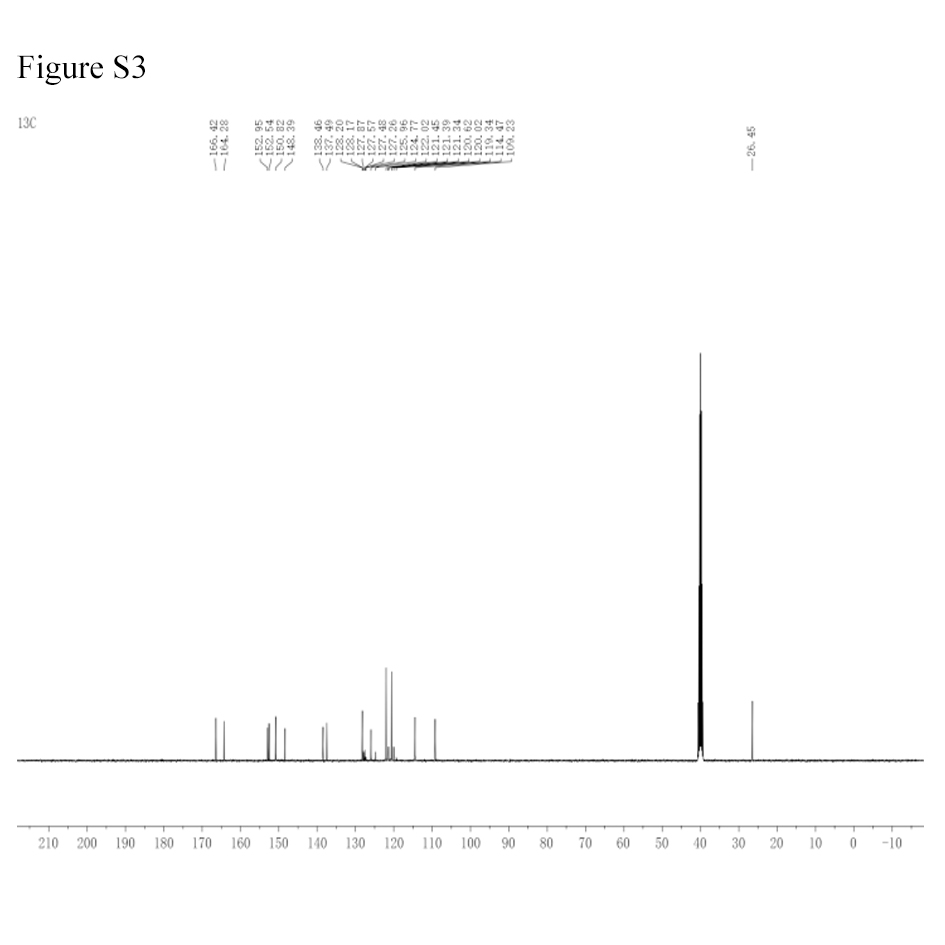

Supplement: Supplementary file 1 [file Image3.jpeg]

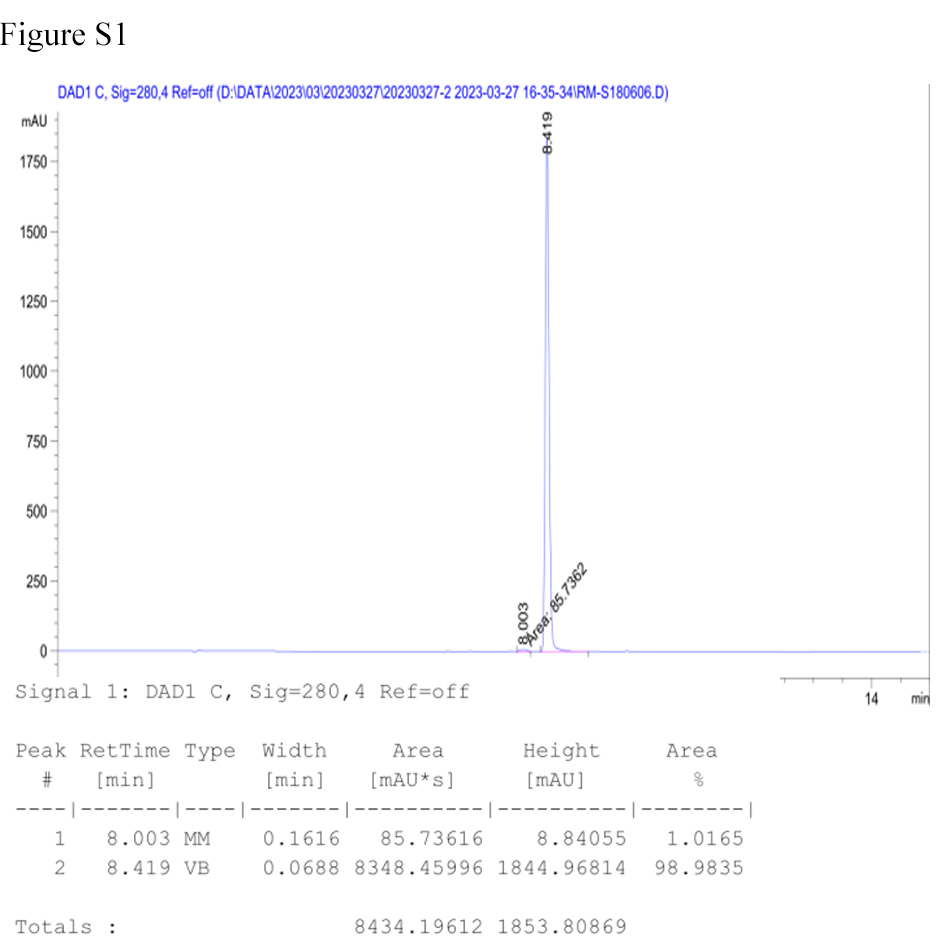

Supplement: Supplementary file 2 [file Image1.jpeg]

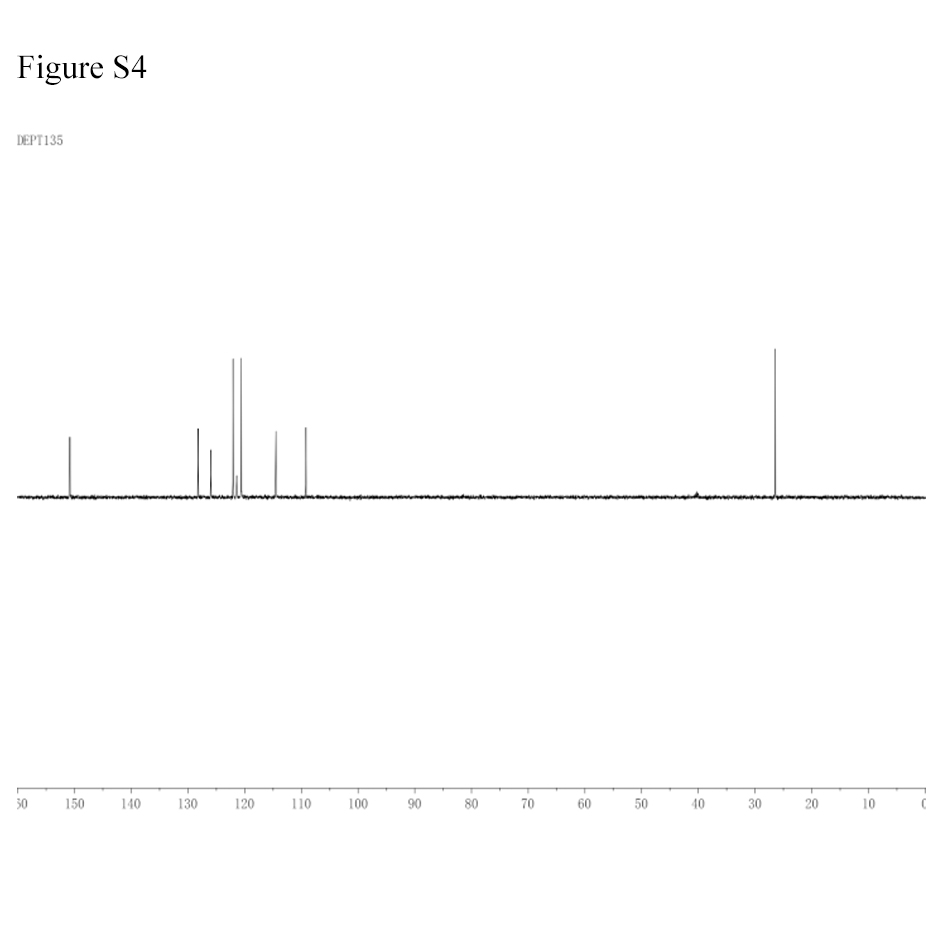

Supplement: Supplementary file 3 [file Image4.jpeg]

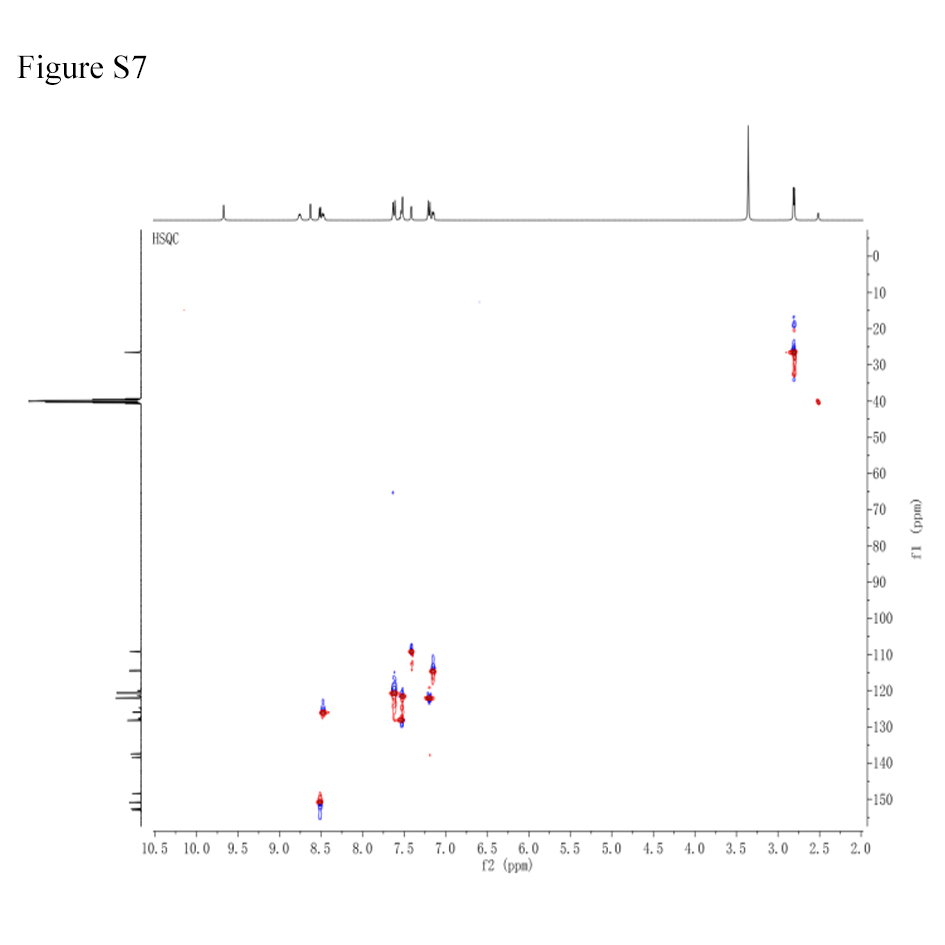

Supplement: Supplementary file 4 [file Image7.jpeg]

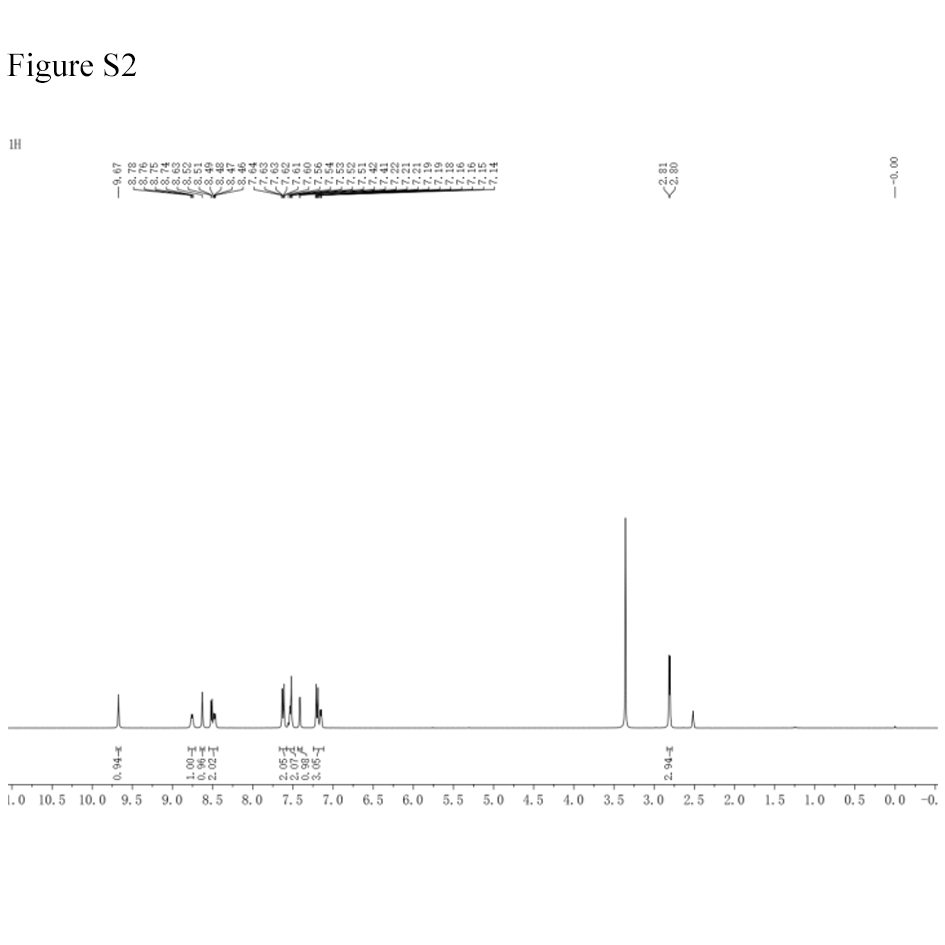

Supplement: Supplementary file 5 [file Image2.jpeg]

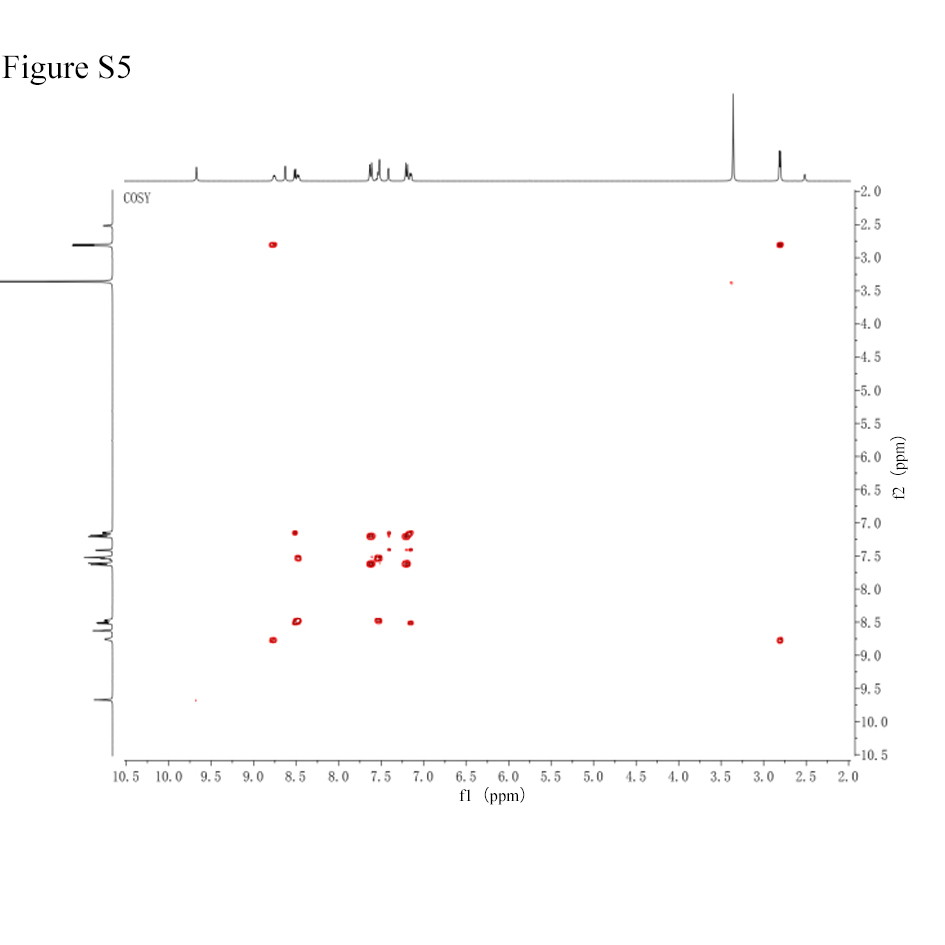

Supplement: Supplementary file 6 [file Image5.jpeg]

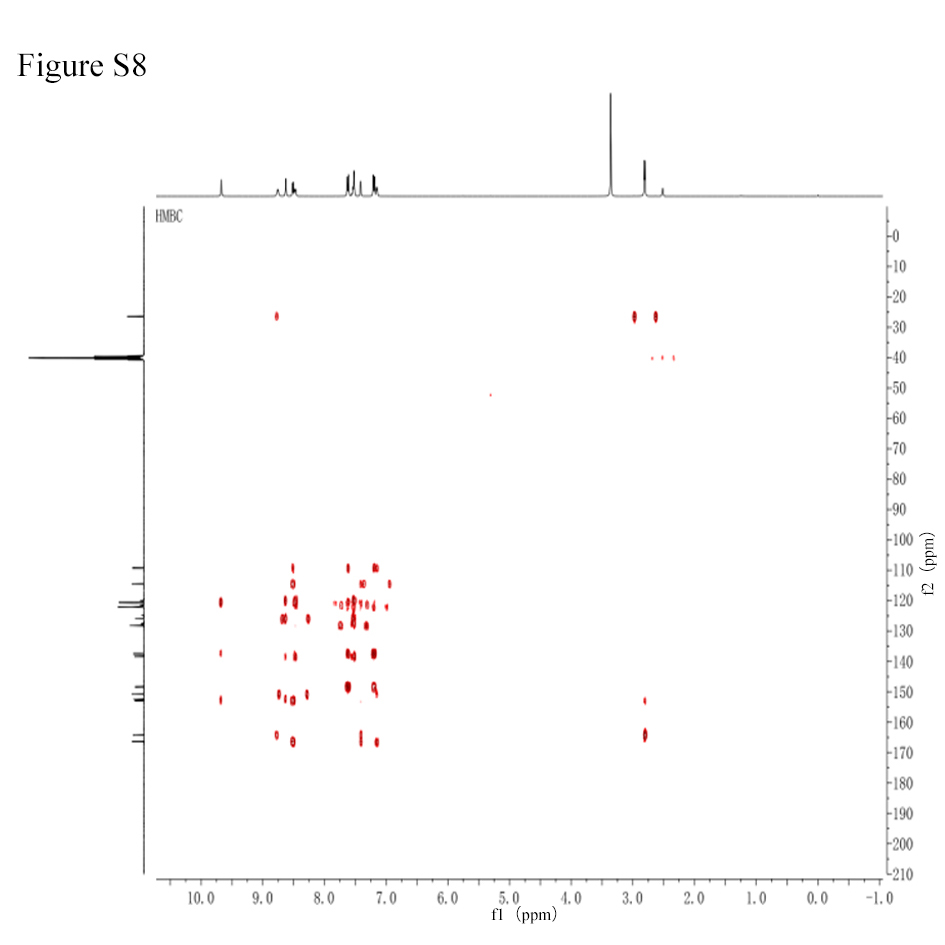

Supplement: Supplementary file 7 [file Image8.jpeg]

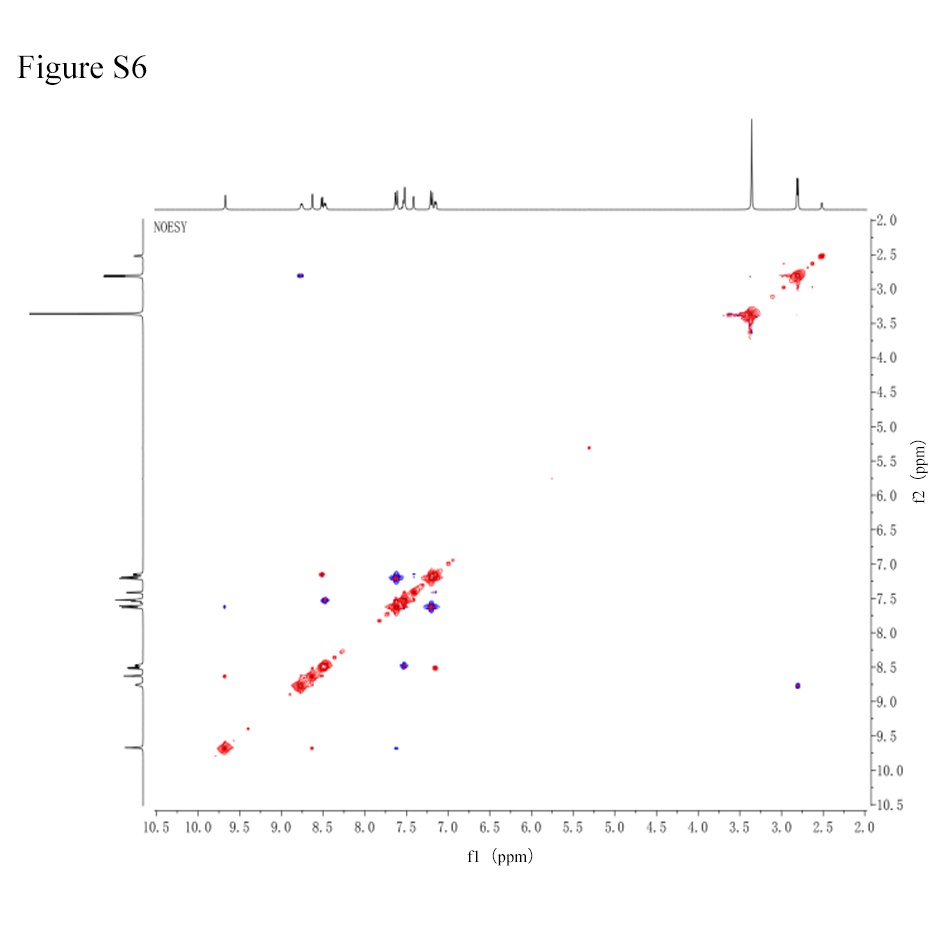

Supplement: Supplementary file 8 [file Image6.jpeg]
